# Functionality of Apps for People with Autism: Comparison between Educators from Florence and Granada

**DOI:** 10.3390/ijerph19127019

**Published:** 2022-06-08

**Authors:** Carmen del Pilar Gallardo-Montes, Antonio Rodríguez Fuentes, María Jesús Caurcel Cara, Davide Capperucci

**Affiliations:** 1 Department of Didactics and School Organization, University of Granada, 51005 Ceuta, Spain; cgallardo@ugr.es; 2 Department of Didactics and School Organization, University of Granada, 18071 Granada, Spain; arfuente@ugr.es; 3 Department of Developmental and Educational Psychology, University of Granada, 18071 Granada, Spain; 4 Department of Education, Languages, Interculture, Literatures and Psychology, University of Florence, 50121 Florence, Italy; davide.capperucci@unifi.it

**Keywords:** autism, ICT, apps, functionality, benefits, applicability, educators

## Abstract

Background: Studies on the potential of smartphone apps for people with autism are currently increasing in number, given the large digital supply available and the benefits they offer. We analyzed the opinion of educators from Florence (Italy) and Granada (Spain) regarding the benefits and applicability of apps, frequency of their use, and the type of apps used for people with autism. Methods: The study involved 1261 professionals, of whom 286 worked with apps, using a non-experimental quantitative design, descriptive and frequency statistics, parametric inferential analyses (Student’s *t* and one-factor ANOVA), and calculation of the effect size (Cohen’s *d* and eta squared) and intrafactorial correlations. Results: Statistically significant differences were observed in respect of city, sex, age, years of experience, place of work, and type of teacher. The teachers from Granada found more benefits and applicability in apps, and revealed a slightly higher usage than those from Florence. Conclusions: It is an arduous but worthy task for professionals from schools and associations that work with people with autism to acquire the necessary knowledge to apply methodologies based on information and communication technology (ICT), as this will help achieve the integrated development of people with different capabilities.

## 1. Introduction

The use of mobile apps is prevalent due to the potential and ease of use they offer, and the convenience and immediacy that they provide. The main app catalogues contain an immense of apps, encompassing a multitude of needs, situations, and target users.

The apps available for smartphones and tablets extend to all sectors of society, and are not confined merely to entertainment use, but address fields such as medicine, education, and social or financial areas.

This proliferation of apps has undoubtedly reached those people with more specific needs and difficulties, such as children and adults with autism. There are hundreds of apps that work in different areas of development (communication, language, basic instrumental skills, executive functions, etc.), and many studies have focused on their potential and the benefits that they provide. One example of this is the research—with encouraging results—on the use of apps focusing on the executive functions for people with autism [1,2,3,4,5,6], on the basic instrumental skills [7,8,9], or on the Theory of Mind [10,11,12,13,14].

However, their use is not as widespread as might be wished. People with autism have difficulties in aspects linked to communication, language, and social interaction [15], and, despite the fact that interventions using information and communication technology (ICT) and apps have produced encouraging results in these areas, such interventions are not particularly common practice in teaching institutions or in psychopedagogical therapies.

The prevalence of people with ASD, at the national and international levels, yields confusing results, since there are no epidemiological studies that reflect the exact number of diagnosed people given the discrepancies in the symptomatology of those who suffer from ASD. Currently, it can be said that there is an increase in diagnoses [15,16,17] because, among other reasons, the procedures are more elaborate [18], the investigations more numerous, and the specialists more varied (physicians, psychologists, pedagogues, speech therapists, etc.). Thus, Fortea et al. [18], in their literature review, state that between 60 and 70 people of every 10,000 present some type of ASD; APA [19] and March et al. [17] show that 1% of the population may have this condition. Anzaldo and Cruz [20] state that it affects 1 in 160 children worldwide. For this reason, the research on autism is abundant [21] and covers different fields of science.

Studies on the usage and frequency of use of apps in formal and informal education with people having autism are, however, non-existent, as is the case with studies on other types of technological resources (Eye Tracking, Virtual Reality, Augmented Reality (AR), Mixed Reality, Robotics, Digital Communication Boards, etc.). There are many studies on the potential of ICT for people with functional diversity and, more specifically, autism [22,23,24,25,26,27], but fewer that look at the motivation for using such resources, the digital training of educators, or the difficulties and simplicities that professionals find in their use. Table 1 shows previous studies from different countries on the perceptions of educators regarding the application of various technologies in the classroom with students with ASD and, specifically, autism.

As stated, research on the motivation for using apps, their frequency of use, and the difficulties found by professionals who work with people with autism when using them are almost non-existent. This is also the case for studies focused on app evaluation. There are several studies on the benefits of using apps designed specifically for people with autism [34,35,36], but not on whether the apps used are of an acceptable quality or if they really attend to the needs and characteristics of users with autism. In this regard, Choez [37] carried out a review and analysis of apps for children with autism, highlighting that, in 2017, there were more than 1300 apps, but there were no studies that assessed whether they were suitable or not. Among the apps analyzed in this study, *José Aprende* and *EmoPLAY* stood out for their quality. Díaz [38] evaluated apps for people with autism, noting the lack of categorizations according to type of disability or the quality of the app, and concluded that only 37.68% were of acceptable quality, and that these focused mainly on the development of social competences. Some of the apps that stood out for their quality were *José Aprende* and *El Viaje de Elisa*. Larco et al. [39] reviewed and assessed 65 apps for people with autism, concluding that 38% were of acceptable quality. These apps (including *Pictoagenda* and *Pictotraductor*) were mainly focused on the development of the social environment, autonomy, and, to a lesser degree, linguistic and communicative skills. Ramón [40] evaluated augmented reality apps, asserting that there was a scarcity of apps designed for this purpose, and offered a list of apps for people with autism, including *#Soyvisual*, *Proyecto Emociones*, and *José Aprende*. Capel [41] analyzed and assessed 50 apps for developing communication for students with autism, indicating that eight were of excellent and 17 of adequate quality. Among the recommended apps were: *#Soyvisual*, *Symbotalk—AAC Talker*, *MITA*, *Pictoagenda*, *Pictotraductor*, and *CommBoards*. Gallardo-Montes et al. [42,43] evaluated 155 apps for people with autism, of which only 14 attained scores that were notably higher than the rest. The apps mainly focused on advancing executive functions, basic instrumental skills, communication, and language, with less space dedicated to emotional development and time management. Noteworthy apps were: *#Soyvisual*, *MITA*, *Symbotalk—AAC Talker*, *CommBoards*, *Smile and Learn*, *LEA*, and *Proyecto Emociones.*

However, as already mentioned, there is a scarcity of research both on the uses made of apps in schools and on psychopedagogical therapies by specialists for attending to people with autism, and on the benefits found from their use. There is not enough information that would make it possible to compare results, or for educators and parents to be able to make a conscious choice over their use. This study, therefore, had the following aims:

1. To investigate the frequency of use of apps by educators in Florence and Granada.
2. To determine the benefits experienced by educators in Florence and Granada when using apps for attending to people with autism.
3. To discover what uses educators in Florence and Granada make of apps for attending to people with autism.
4. To determine the benefits and uses of apps according to sex, age, years of experience, city of origin, place of work, and type of teacher.
5. To identify the apps that are specifically used for people with autism by educators in Florence and Granada.

## 2. Method

This was a quantitative study with a non-experimental, comparative, and cross-sectional descriptive design.

### 2.1. Participants

The study sample comprised 286 professionals from the education sector, from the cities of Florence (F), Italy (*n* = 159), and Granada (G), Spain (*n* = 127), who reported using apps for the care of people with autism. In the selection of participants, it was essential that they had experience in the special education sector and, specifically, that they work or had worked with people with autism.

For the participant selection, non-probability convenience sampling was undertaken. Educators participated on a voluntary basis, without receiving any incentive.

The participants from Granada consisted of 159 women (84.3%) and 25 men (15.7%), with ages between 20 and 64 years old (*M* = 37.67; *SD* = 10.50), and with experience working with children with autism below the age of 10 years old (84.9%). The majority were Therapeutic Pedagogy teachers (34.6%) and general teachers (20.1%) who worked in the stages of primary education (80.5%) and preschool (57.2%), and in schools with Internet access (98.7%) and available computers (93.7%), tablets (73.6%) and projectors (62.3%).

The participants from Florence were made up of 127 women (89.9%) and 13 men (10.2%), aged between 25 and 58 years old (*M* = 37.98; *SD* = 8.14), and with experience with people with autism younger than 10 years old (95.3%). They were mostly support teachers (74.0%) and general teachers (42.5%) who worked in the stages of primary education (55.9%) and preschool (37.8%), and in schools with Internet access (95.3%), and with computers (89.8%), projectors (56.7%), and tablets (55.9%).

Bearing in mind the significant number of female participants, the sex and/or gender did not result in bias in this investigation, as studies in Social Science and Legal Science have a predominance of women [44,45].

### 2.2. Instrument

In order to discover the use of the apps by the educators, the frequency of that use, and the benefits they experience in the apps’ use when working with people with autism, we administered the Italian version of the questionnaire—“Questionario sulla formazione e sulle competenze legate all’use delle ICT degli insegnati che operano con alunni disabili” [“Questionnaire on training and competences related to the use of ICT for teachers who work with disabled pupils”] [46], and the Spanish version, “Demandas y potencialidades de las ICT y las apps para la atención a personas con autismo (DPTIC-AUT-Q)” [“Demands and Potentials of ICT and Apps for Assisting People with Autism”] [47]. This questionnaire has a section on sociodemographic data and four subscales connected to ICT: Subscale 1: Opinion, training, and uses of ICT by professionals for teaching people with functional diversity; Subscale 2: Training and uses of ICT by professionals for teaching people with autism; Subscale 3: Uses and benefits of apps in assisting people with autism; Subscale 4: Uses and possibilities of specialized apps for people with autism. In order to meet the aims of this study, we only used the third subscale, “Uses and benefits of apps in assisting people with autism”, which comprised questions with Likert-scale responses (1 = Completely disagree; 5 = Completely agree). It had two dimensions: Dimension 1 (D1): Benefits of apps for autism (items 63–72); Dimension 2 (D2): Uses of apps for autism (items 73–84), and a question on the frequency of use of apps (1 = Little, 4 = A lot).

The questionnaire has adequate psychometric properties. It obtained excellent intraclass correlation coefficients in Subscale 3 (Italian version = 0.955; Spanish version = 0.994); significant Kendall’s *W* inter-rater concordance, *p* < 0.001 (Italian version = 0.192 clarity; 0.197 coherence; 0.202 relevance; and 0.218 objectivity; Spanish version = 0.138 clarity; 0.132 coherence; 0.155 relevance; and 0.123 objectivity); and exceptional internal consistency: *α_Italy_Subscale_3_* = 0.998; *α_Spain_Subscale_3_* = 0.951.

### 2.3. Procedure

First, the approval of the University of Granada Ethics Committee on Human Research (Spain) was obtained for the questionnaire, for which a favorable report was received [2002/CEIH/2021]. The questionnaire was then administered during the period between 2020 and 2021. In the case of Italy, the questionnaire was administered to teachers who were undergoing sessions of continuing education for teachers attending to diversity, at the Università degli Studi di Firenze (University of Florence). In Spain, the questionnaire was administered to teachers and educators of schools and associations in the city of Granada. The link to access the questionnaire, designed on the *LimeSurvey* platform, was provided in face-to-face sessions and via email. At all times, participants were informed of the voluntary nature of the questionnaire, its anonymity and data exclusivity, and the aims of the study.

### 2.4. Data Analysis

The data were analyzed with the SPSS v.26.0 statistics software. The descriptive statistics (mean, mode, and standard deviation), frequencies, parametric inferential and intrafactorial correlation analyses were calculated. For the dichotomous variables “city”, “sex”, and “type of teacher in Granada”, Student’s *t* test was used and the effect size calculated (Cohen’s *d*). For the variables “years of experience with autism”, “age”, “educational stage”, “place of work”, and “type of teacher in Florence”, the ANOVA *F*-test was used, followed by Tukey’s HSD and Bonferroni tests, along with the homogeneous subsets test, calculating the effect size using eta squared (*η*^2^). Estimations of the effect size were also performed by calculating Cohen’s *d* and eta squared (small: 0.20 < *d* < 0.30; medium: 0.30 < *d* < 0.80; and large: *d* > 0.80).

For the data analysis relative to the variable “age”, four groups were established according to the minimum and maximum age of the participants: Group 1: 20 to 30 years old; Group 2: 31 to 40 years old; Group 3: 41 to 50 years old; and Group 4: 51 to 64 years old. Regarding the variable “years of experience”, the groups established were: Group 1: ≤5 years; Group 2: 6–10 years; Group 3: 11–20 years; and Group 4: 21–30 years. The option >30 years of experience with people with autism was covered in the questionnaire, but no participant had such professional experience, and so this group was not included in the analyses.

## 3. Results

The frequency of use of apps (Table 2) showed that the teachers in Granada used them to a greater extent than their Florentine counterparts. In Granada, the option “quite a lot” was chosen by 50.3% of the participants, compared to 44.9% in Florence.

In Table 3, according to the values of the mean and the mode, we can see that the opinion of the participants on the benefits and uses of the apps for people with autism was situated between Options 4 “Agree” and 5 “Fully agree”. Only Item 72, “Enhances socialization”, was positioned at Option 3, “Neither agree nor disagree”.

In Dimension 1, regarding the benefits that apps can provide people with autism, the educators showed that the apps proved to be a motivating option for working with them, since they complemented the use of other, more traditional media (books, blackboard, etc.), and helped to reinforce and consolidate the concepts previously studied. However, they were less in agreement that apps enhanced socialization, learning to read, and the encouragement of leisure and entertainment.

Regarding Dimension 2, concerning the uses the educators made of the apps with people with autism, the most notable feature was that they helped to hold attention for longer, and they facilitated communication, development of autonomy, and learning calculus. However, their use was not aimed at the development, understanding, and expression of emotions, or at tasks related to time management or self-regulation.

Table 4 shows how the participants from Granada yielded a higher mean than those from Florence with regard to the benefits that they considered the apps provided for people with autism (Dimension 1), and the uses they made of them (Dimension 2).

The analysis of correlations (Table 5) showed that there was a strong (*r* = 0.795) and significant (*p* < 0.01) positive relation between the benefits that the educators found in apps and the use and utility they assigned them. Significant (*p* < 0.01) but weak (from 0.297 to 0.311) positive correlations can similarly be seen in the frequency of use of apps with the benefits (*r* = 0.297) and with the usefulness (*r* = 0.311) they assigned them.

Weak (from −0.080 to −0.126) negative correlations can be seen between the age of the participants and the benefits (*r* = −0.126) and uses of the apps (*r* = −0.080).

According to the city of origin (Table 6), significant differences can be observed in 36.36% of the items (*p* < 0.05). In Dimension 1 (D1), regarding the benefits of the apps, the participants from Granada obtained higher means than the teachers from Florence, with an effect size between small (*d* > 0.20) and medium (*d* > 0.50). These items indicate that the apps for people with autism stimulated cognitive development (*t*(284) = −2.57, *p* = 0.011), enabled learning how to read (*t*(284) = −2.33, *p* = 0.021), promoted leisure and entertainment (*t*(284) = 5.37, *p* = 0.000), and, moreover, were a motivating tool for working with them (*t*(284) = 2.94, *p* = 0.004).

In Dimension 2 (D2), regarding the uses of the apps by the educators, the participants from Granada similarly obtained higher means than those from Florence, with an effect size between small (*d* > 0.20) and medium (*d* > 0.50). The differences showed that the teachers from Granada used apps more with the purpose of developing communication (*t*(284) = −2.37, *p* = 0.018), oral language (*t*(284) = −2.19, *p* = 0.029), comprehension (*t*(284) = −3.19, *p* = 0.002), and expression of emotions (*t*(284) = −3.41, *p* = 0.001), than the teachers from Florence.

The variable “sex” produced significant differences (Table 7) in two items of Dimension 2, with a medium effect size (*d* > 0.50). The women from Florence used apps more than the men for the purpose of holding the attention of people with autism for longer (*t*(125) = −2.25, *p* = 0.037). The women from Granada used apps for developing the understanding of emotions more than men (*t*(157) = −2.13, *p* = 0.034).

The variable regarding the age of the participants revealed differences in the teachers from the two countries (Table 8), with more differences arising among those from Granada.

In Florence, the oldest participants (51 and 64 years old) were those who used apps the least for developing autonomy (*F(3)* = 2.84, *p* < 0.041, *η*^2^ = 0.058) (item 78-D2) compared to their younger colleagues.

In Granada, the youngest teachers (20–30 years old) held the view—to a greater degree than the rest—that the benefits found in apps were that they made psychopedagogic intervention more effective (*F(3)* = 2.84, *p* < 0.38, *η*^2^ = 0.019) and that they consolidated concepts (*F(3)* = 2.90, *p* < 0.037, *η*^2^ = 0.046) (items 68 and 70-D1). In terms of using apps for developing communication (*F(3)* = 3.37, *p* < 0.013, *η*^2^ = 0.007) (item 73-D2), those aged between 41 and 50 years old stated that they used them more, compared to those aged between 31 and 40 years old. With regard to using apps to carry out tasks of planning (*F(3)* = 2.98, *p* < 0.033, *η*^2^ = 0.035) and organization (*F(3)* = 2.88, *p* < 0.038, *η*^2^ = 0.045) (items 79 and 80-D2), the teachers aged between 41 and 50 years old reported using them more than the other participants. Lastly, regarding the use of apps for carrying out tasks related to self-regulation (*F(3)* = 3.60, *p* < 0.015, *η*^2^ = 0.049) (item 81-D2), the youngest age group (20–30 years old) presented a greater use than the teachers from Granada aged between 31 and 40 years old.

As a function of years of experience, we only found significant differences for the participants from Florence (Table 9): *F(3)* = 3.83, *p* = 0.012, *η*^2^ = 0.019. The Bonferroni and Tukey post hoc comparisons and the homogeneous subset tests revealed that the educators with 11 to 20 years’ experience made more use of apps for the development of communication for people with autism (*M* = 5.00, *SD* = 0.00), as opposed to those with 6 to 10 years’ experience (*M* = 3.39, *SD* = 0.96) (item 73-D2).

The educational stage taught by the teachers also proved to be a determining factor (Table 10). In Florence, those working in primary education considered that it was more beneficial to work with apps to enable people with autism to learn how to read than those teachers from preschool education (*F(3)* = 2.76, *p* < 0.045, *η*^2^ = 0.100) (item 65-D1). Those in secondary education found more benefits than the teachers who simultaneously worked in both preschool and primary education in the use of apps to consolidate concepts (*F(3)* = 3.11, *p* < 0.029, *η*^2^ = 0.026) (item 70-D1), to promote socialization (*F(3)* = 2.94, *p* < 0.036, *η*^2^ = 0.012) (item 72-D1), and to hold attention for longer (*F(3)* = 6.22, *p* < 0.001, *η*^2^ = 0.069) (item 84-D2). The teachers in both preschool and primary education simultaneously revealed that they most agreed with using apps to carry out tasks related to self-regulation (*F(3)* = 3.03, *p* < 0.032, *η*^2^ = 0.066) (item 82-D2) and learning calculus (*F(3)* = 4.30, *p* < 0.006, *η*^2^ = 0.067) (item 83-D2) than those who only worked in primary education. Similarly, the participants from secondary education agreed most with the use of apps to learn calculus compared to the preschool teachers.

In Granada (Table 11), the main differences can be observed in the teachers of primary education over those from secondary education in revealing a greater degree of agreement on the benefits of apps for encouraging leisure and entertainment (*F(4)* = 3.33, *p* < 0.012, *η*^2^ = 0.022) (item 66-D1), for the motivation these tools give (*F(4)* = 3.21, *p* < 0.014, *η*^2^ = 0.008) (item 71-D1), and the good use they can be put to in order to hold student attention for longer (*F(4)* = 2.98, *p* < 0.021, *η*^2^ = 0.027) (item 84-D2). The educators who worked with adults also showed a higher degree of agreement than those from secondary education regarding the benefits of apps for carrying out memory-based tasks (*F(4)* = 2.76, *p* < 0.032, *η*^2^ = 0.006) (item 64-D1), the motivation they give the student (*F(4)* = 3.21, *p* < 0.014, *η*^2^ = 0.008) (item 71-D1), and their use for learning calculus (*F(4)* = 2.84, *p* < 0.021, *η2* = 0.027) (item 84-D2).

The teachers’ work location (urban/rural/both) produced statistically significant differences in both countries. We can see that the Florentine teachers (Table 12) from urban areas revealed more benefits in using apps to enhance socialization than the teachers from rural areas or with jobs in both places (*F(2)* = 3.31, *p* < 0.040, *η*^2^ = 0.007) (item 72-D1). A similar result was found with the use of apps for developing communication (*F(2)* = 3.45, *p* < 0.035, *η*^2^ = 0.025) (item 73-D2) and expressing emotions (*F(2)* = 3.73, *p* < 0.027, *η*^2^ = 0.020) (item 76-D2).

The participants from Granada (Table 12) who worked in both locations (rural and urban) found more benefits than those who were only in either rural or urban areas in using apps as a complement to reinforce what had previously been worked on (*F(2)* = 4.40, *p* < 0.014, *η*^2^ = 0.040) (item 69-D1).

The work location revealed differences in the participants from the two cities. The general teachers from Florence (Table 13) showed more agreement over the use of apps for developing oral language, compared to those who worked as both general and support teachers (*F(2)* = 4.47, *p* < 0.013, *η*^2^ = 0.07) (item 74-D1).

The general teachers from Granada (Table 14) were more in agreement, with a medium effect size (*d* ≥ 0.50), regarding the use of apps as a way to enhance learning how to write, compared with the support teachers (Therapeutic Pedagogy) (*t*(85) = 2.13, *p* = 0.037) (item 82-D2).

The apps specifically for people with autism shown in Table 15 are ordered according to the ranking of the best apps established by Gallardo-Montes et al. (2021). In this ranking, the highest-rated and highest-quality apps at national and international levels are positioned in descending order.

The teachers from Florence (*N* = 127) did not reveal a significant use of apps specifically for people with autism; only three of them were highly used of the 23 that were presented: *MITA: Language and Cognitive Therapy* (*n* = 27) (focused on working with the basic instrumental skills, executive functions, and leisure and entertainment) (languages: Italian, English, and Spanish); *Aboard CAA (ACC)* (*n* = 26) (language: English), and *Symbotalk—AAC Talker* (*n* = 18) (both focused on developing communication, language and the executive functions) (languages: Italian, English, and Spanish).

The educators from Granada (*N* = 159) revealed a greater use of specialized apps. Of the 23 apps set out, 12 were used the most: *#Soyvisual* (*n* = 80) (language: Spanish); *Smile and Learn: Juegos educativos para niños* (*n* = 73) (languages: Italian, English, and Spanish); *LEA: lecto escritura para autismo* (*n* = 69) (language: Spanish); *José Aprende* (*n* = 69) (language: Spanish); *¡Emociones, sentimientos y expresiones!* (*n* = 57) (language: English); *Proyecto emociones* (*n* = 44) (language: Spanish); *Proyect@ PECS* (*n* = 40) (language: English); *MITA: Language and Cognitive Therapy* (*n* = 32) (languages: Italian, English, and Spanish); *Symbotalk—AAC Talker* (n = 45) (languages: Italian, English, and Spanish); *Juego de niños para bebés de 2 a 5 años* (*n* = 38) (language: English); *Aboard CAA (ACC)* (*n* = 23) (language: English); and *Lista visual -Visual Schedule* (*n* = 21) (languages: Italian, English, and Spanish). These apps were designed for developing communication, language, and the emotions (Theory of Mind), enhancing the basic instrumental skills, executive functions, time management, and leisure and entertainment.

Statistically significant differences were revealed in the use of specialized apps for people with autism according to the city of origin (Table 16).

The educators from Florence were different in the use of two specialized apps compared to those from Granada: *Juego de niños para bebés de 2 a 5 años* (language: English) (*t*(284) = −5.13, *p* = 0.000) and *Vi.co hospital Lite* (languages: Italian, English, and Spanish) (*t*(284) = 2.77, *p* = 0.006) with a small (*d* ≥ 20) and medium (*d* ≥ 0.50) effect size, respectively.

The participants from Granada revealed that they made greater use of 10 specialized apps compared to those from Florence. Those with a large effect size (d ≥ 0.80) are: *#Soyvisual* (language: Spanish) (*t*(284) = −10.99, *p* = 0.000), *LEA: lecto escritura para autismo* (language: Spanish) (*t*(284) = −9.25, *p* = 0.000), and *José Aprende* (language: Spanish) (*t*(284) = −9.83, *p* = 0.000). The apps with a medium effect size (*d* ≥ 0.50) are: *¡Emociones, sentimientos y expresiones!* (language: English) (*t*(284) = −7.28, *p* = 0.000), *Proyecto emociones* (language: Spanish) (*t*(284) = −6.08, *p* = 0.000), *Proyect@ PECS* (language: English) (*t*(284) = −4.37, *p* = 0.000), and *Lista visual -Visual Schedule* (languages: Italian, English, and Spanish) (*t*(284) = −4.38, *p* = 0.000), whereas the following apps show a small effect size: *Smile and Learn: Juegos educativos para niños* (languages: Italian, English, and Spanish) (*t*(284) = −8.93, *p* = 0.000), *Symbotalk—AAC Talker* (languages: Italian, English, and Spanish) (*t*(284) = −2.90, *p* = 0.000), and *Autastico* (language: English) (*t*(284) = −1.98, *p* = 0.000).

## 4. Discussion and Conclusions

ICT offers a world of possibilities that, combined with responsible usage, can encourage and enhance cognitive processes and the development of different capabilities in people with functional diversity. More specifically, it has been shown that apps are a motivating tool [30] and a good teaching option for working with people with autism [29], but their use is not as widespread as it should be.

The frequency with which the participants used the apps lies between the options “sometimes” and “quite a lot”, with a slightly higher frequency observable for the teachers from Granada. The Spanish participants found more benefits and greater applicability in apps for attending to people with autism than their Florentine counterparts.

Generally speaking, the participants from both countries found that the apps were beneficial because they could be used as a complement to traditional media and methods, in order to consolidate and reinforce concepts previously worked on and, furthermore, as a motivating resource, a finding that is in line with Martínez [30]. Fewer benefits were found in the use of apps for the learning of reading and for promoting socialization and leisure. Regarding the uses that the teachers made of the apps, these were mostly as a means to hold attention for longer, to enhance communication, to develop autonomy, and to encourage the learning of calculus. A lesser use of apps was connected to the development and expression of emotions, and to carrying out tasks connected to time management, organization, planning, and self-regulation.

Strong correlations, therefore, were found between the benefits and the use of apps. As more benefits were found after using the apps, the educators discovered greater applicability in them for work and attending to people with autism. We also observed that the higher the frequency of app use, the more benefits and applicability the participants from both countries found in them. This suggests that, previously, the potential of the apps may not be perceptible, and the apps designed specifically for working with people with autism may not be known [29], or there is a lack of training for their use [29,30,31]; however, as they are used, the return and practice increase. Teachers need to know the digital resources that are suitable for each student [33], in order to be able to use them and give meaning to their educational practice.

As a function of the city of origin, the participants from Granada found more benefits than those from Florence in the apps as a motivating tool for cognitive development, learning how to read, and the promotion of leisure and entertainment. Equally, the Granada teachers revealed a greater use of apps for developing communication [33], oral language, and the understanding and expression of emotions. These results are curious, as there are more differences than expected according to this variable. It would be interesting to investigate these aspects in future studies, complementing this quantitative research with individual in-depth interviews.

In terms of sex, Florentine women used apps more than the men for the purpose of holding attention for longer when attending to people with autism. However, in Granada, the female participants used the apps more to develop understanding of emotions. There are no previous studies regarding this variable, nor on the use of apps; therefore, no comparisons can be made.

The variable of age also produced differences according to country. In Florence, the oldest participants used apps less for developing autonomy in people with autism than their younger colleagues. In Granada, the youngest teachers found more benefits in the use of apps to consolidate concepts and to make psychopedagogical interventions more effective. The participants aged between 41 and 50 years old revealed a greater use of apps for developing communication and to carry out tasks linked to planning and organization when compared to those aged between 31 and 40 years old. These data cannot be compared to previous studies: as stated before, there are no studies that examine these aspects.

According to the variable of the teachers’ years of experience with people with autism, although in Granada there were no apparent differences, this was not the case in Florence. The participants with less experience indicated that they used apps for developing communication less than those with more experience. In the study by Saladino et al. [33], in Italy, it was found that educators who had more professional experience with people with ASD used ICT less frequently. Nevertheless, a priori, these results could be compared due to the similarity of the variable “experience” and “city of origin”, as the data are very generic and do not provide detail about the use of apps or the purpose of using ICT.

Statistically significant differences were also found according to the education stage. In Florence, the teachers of primary education were more aligned with those from preschool education regarding the benefits of apps to facilitate learning how to read. The participants who taught secondary school found more benefits and greater applicability in apps as a socializing tool, for consolidating concepts, for learning calculus, and for holding attention for longer when teaching people with autism, compared to the earlier educational stages. Those participants who worked in both preschool and primary education were more in agreement over the use of apps for developing writing than those who only taught the preschool stage. In contrast to Florence, in Granada, the educators who worked with adults and in primary education stated that apps were a motivating tool and that they helped to hold students’ attention for longer, in comparison with the participants who worked in secondary education. The main difference occurred in Item 84, regarding the use of apps to hold the attention of people with autism for longer. Whereas in Florence the secondary school teachers were more in agreement over this item than those from preschool and primary education, in Granada it was those from primary education who stood out over those from secondary education.

The location of the teachers’ employment also had an influence. In Florence, the participants from urban areas were more in agreement than those from rural areas over the use of apps to enhance socialization, and to develop communication and expression of emotions. However, the teachers from Granada who worked in both rural and urban areas indicated that they were more aligned over the benefits of apps as a complement to reinforce what had been worked on previously.

Regarding the job description, in Florence, those who worked as general teachers showed greater agreement on the use of apps for developing oral language. This was also the case in Granada, but in regard to the use of apps for learning how to write.

The use of specialized apps revealed higher use by the teachers from Granada compared to their Florentine counterparts. They were presented with a list of 23 specialized apps that stood out for their quality in catering for people with autism. Only three of these apps had a high usage by the participants from Florence, which were the same apps mentioned in previous studies on the benefits apps provide this group (*MITA: Language and Cognitive Therapy*, *Aboard CAA (ACC)*, *and Symbotalk—AAC Talker*) [35,36,37]. In Granada, there was a more positive tendency. The percentage of apps used was higher, as were the participants who used them. Twelve apps were used the most: *#Soyvisual*, *Smile and Learn: Juegos educativos para niños*, *LEA: lecto escritura para autismo*, *José Aprende*, ¡*Emociones, sentimientos y expresiones!*, *Proyecto emociones*, *Proyect@ PECS*, *MITA: Language and Cognitive Therapy, Symbotalk—AAC Talker, Juego de niños para bebés de 2 a 5 años*, *Aboard CAA (ACC)*, and *Lista visual -Visual Schedule*. These results are encouraging, since they mean that the different specialists in autism in the province of Granada support teaching and psychopedagogical therapy with validated digital tools that had the quality required for the needs of people with this disorder. These apps can also be found in previous studies, such as Aguilar-Velázquez et al. [9], Capel [41] Choez [37], Díaz [38], Gallardo-Montes et al. [42,43], Ramón [40], and Vyshedskiy et al. [36].

The language of the apps was not a deciding factor for their use. Most of them were in English, yet, despite this, they were used more by participants from Granada than from Florence. There were statistically significant differences in the use of some apps that were only in Spanish and not in Italian; thus, the reason for the differences in favor of the teachers from Granada was understandable. This only occurred with four apps of the ten that revealed differences, since the rest were in Italian, English, and Spanish. For this reason, it should be repeated that language was not a determining factor.

The teachers from both cities highlighted the benefits of apps for cognitive stimulation and their preferred use for developing communication and oral language. These aspects are priorities in the progress of people with autism, as a fundamental part of their comprehensive development. These results fit perfectly with the aforementioned apps, since they were mainly focused on communicative-linguistic development and the executive functions. It was also evident that the apps were used very little for the understanding and expression of emotions, time management, planning, and organization, which may be connected to the scarcity of apps aimed at developing the Theory of Mind and time management for people with autism [43]. These results may also be due to the teachers’ lack of digital training [29,30,31], or the insufficient funding and resources available in formal and informal education [31].

Among the benefits indicated by the teachers, it was highlighted that apps complement the use of other work media such as textbooks or the blackboard, that they are motivating tools for people with autism, and that they are an aid to reinforcing and consolidating concepts. In summary, the apps significantly support the development of key competences [33], enable access to the school curriculum [32], and provide assistance during cognitive therapies [31].

For future research, it will be important to address the subject of teachers’ digital training regarding the existing resources for working with people with autism (apps, Eye Tracking, Augmented Reality (AR), Digital Communication Boards, etc.), thus further examining whether different professionals have sufficient training in ICT or if their schools’ funding is sufficient for using technologies in classrooms or therapy. Similarly, it would be enriching to continue researching in other international contexts on the digital training of the different educators who attend to people with autism, or even to carry out experimental studies in which the digital competence of educators is developed in order to make better use of these digital tools with people with autism. In the methodological dimension, the invariance of measurement of the instrument in the two languages needs to be examined, following the proposal by [48]. This would provide more consistency to the contrast between constructs and items of the two groups of teachers, i.e., Spanish and Italian. Along the same methodological lines, it would have been interesting to further the analyses carried out using multivariate analysis of variance, in order to observe the behavior of the elements in an interrelated manner.

Among the limitations of this study is the difficulty in accessing a larger sample, because the use of apps is not very widespread in education and in attending to people with autism. Another limitation is tied to the lack of previous studies with which to compare our results in terms of age, sex, and professional experience with people with autism.

The practical implications derived from this study are centered on showing the benefits and didactic applications of the apps for people with autism, and the specific uses that different specialists make of them at an international level. Identifying the professional perception of these digital resources is essential for guiding current educational practices toward more innovative and flexible learning models.

## Figures and Tables

**Table 1:** Previous studies on the perception of teachers regarding the application of technologies in the classroom with people with autism.

Study	Sample	Objectives	Conclusions
[28]	Seven teachers (Mexico)	To analyze the impact of implementing MOBIS (Augmented Reality—AR) with teachers of students with autism	- AR is a real support aid during cognitive therapy - It improves teaching performance - It reduces teachers’ workload
[29]	20 schools (Spain)	Identify and assess the application of inclusive response measures in classrooms specifically for students with ASD	- Apps show potential for working with students with ASD - Lack of teacher training in technologies - Lack of knowledge about apps for students with ASD
[30]	20 teachers (Spain)	Analyze teacher training with regard to ICT and AR in specialized classrooms	- ICT and AR are a motivating element for teaching - ICT and AR promote active participation - Lack of teacher training on digital technology
[31]	70 teachers (Jordan)	Identify the obstacles teachers find when implementing technologies in classrooms with students with autism	- Lack of equipment, digital resources, and coordination between teachers - Lack of training for designing digital educational programs - Difficulty of students in using different technologies - Software barely adapted to the needs of students with autism
[32]	Eight teachers (Malaysia)	Perception of teachers on using the app *Autism Aid* in classrooms with students with autism	- The most experienced teachers support the use of tablets with apps more than those with less experience - Most teachers recognize the potential of tablets - Tablets can disrupt the pace of the class and the rest of students - Apps facilitate access to the school curriculum
[33]	142 teachers (Italy)	Discover teachers’ perception about the use of technologies	- Technologies significantly assist in the development of key competences - They encourage the participation and inclusion of students with ASD - Apps enhance the communication and interaction of children with ASD - Teachers with more experience do not use ICT - Teachers need to know which digital resources are suitable for each student

**Table 2:** Frequency of use of apps in Granada and Florence (*N* = 286).

Frequency of Use	Granada *N* (%)	Florence *N* (%)
Little	5 (3.1)	7 (5.5)
Sometimes	64 (40.3)	51 (40.2)
Quite a lot	80 (50.3)	57 (44.9)
A lot	10 (6.3)	12 (9.4)

**Table 3:** Opinion of teachers from Granada and Florence about the benefits and uses of apps for attending to people with autism (*N* = 286).

ITEM		*M*	*SD*	*M_o_*	%				
					1	2	3	4	5
**D1. Benefits of apps**	63. Stimulate cognitive development	4.15	0.83	4	1.4	2.4	11.9	48.6	35.7
	64. Facilitate performing tasks related to memory	4.07	0.88	4	1.0	4.2	16.4	43.4	35.0
	65. Enable learning how to read	3.96	0.96	4	2.8	3.8	19.9	41.6	31.8
	66. Promote leisure and entertainment	3.85	1.08	4	3.8	8.4	17.8	38.5	31.5
	67. Complement the use of other work media...	4.30	0.91	5	2.1	3.5	7.7	35.7	51.0
	68. Make the psychopedagogic intervention...	4.05	0.90	4	1.4	4.2	16.8	43.4	34.3
	69. It is a good complement to reinforce what has been worked…	4.21	0.86	5	1.4	2.8	11.9	41.6	42.3
	70. It is a way to consolidate concepts	4.22	0.76	4	0.3	1.7	12.6	46.5	38.8
	71. It is a motivating tool	4.40	0.78	5	0.3	2.4	9.1	32.9	55.2
	72. It facilitates socialization	3.46	1.02	3	2.8	13.6	36.0	29.7	17.8
**D2. Uses of apps**	73. Develop communication	3.92	0.90	4	2.1	4.2	19.6	47.9	26.2
	74. Develop oral language	3.74	0.99	4	3.1	7.0	25.5	41.3	23.1
	75. Develop understanding of emotions	3.71	1.01	4	4.5	4.9	26.9	42.0	21.7
	76. Develop the expression of emotions	3.67	1.02	4	3.8	7.7	27.3	39.5	21.7
	77. Manage time	3.63	1.06	4	3.8	10.1	27.6	36.4	22.0
	78. Develop autonomy	3.91	0.94	4	2.4	4.5	21.0	43.7	28.3
	79. Carry out tasks related to planning	3.77	1.00	4	3.8	5.6	24.5	42.0	24.1
	80. Carry out tasks related to organization	3.78	0.99	4	3.1	6.6	24.1	41.6	24.5
	81. Carry out tasks related to self-regulation	3.71	1.00	4	4.2	4.9	29.4	39.2	22.4
	82. Facilitate learning how to write	3.76	1.07	4	3.8	9.4	20.6	38.8	27.3
	83. Enable learning calculus	3.81	1.04	4	4.9	6.3	17.1	46.2	25.5
	84. Hold attention for longer	4.20	0.82	4	0.7	2.8	13.3	42.3	40.9

Note. *M* = Mean; *SD* = Standard deviation; *M_o_* = Mode.

**Table 4:** Mean and standard deviation of the benefits and uses of apps according to each city.

City	Benefits Apps		Uses Apps	
	*M*	*SD*	*M*	*SD*
Granada (*n* = 159)	4.14	0.65	4.65	0.86
Florence (*n* = 127)	3.97	0.67	4.45	0.91
Total (*n* = 286)	4.07	0.66	4.56	0.89

Note. *M* = Mean; *SD* = Standard deviation.

**Table 5:** Pearson’s correlation coefficient between the benefits of the apps, the uses and applications, the age of the educator, and the frequency of use.

	Benefits	Uses	Age	Frequency
1. Benefits of the apps	1			
2. Uses and applications of apps	0.795 **	1		
3. Age of the educator	−0.126 *	−0.080	1	
4. Frequency of use of apps	0.297 **	0.311 **	0.002	1

Note: ** The correlation is significant at level 0.01 (two-tailed); * The correlation is significant at level 0.05 (two-tailed).

**Table 6:** Significant differences in the benefits and uses of apps as a function of city.

	Dependent Variables	Florence		Granada		*t*	*d*
		*M*	*SD*	*M*	*SD*		
**D1**	63. Stimulate cognitive development	4.01	0.87	4.26	0.77	−2.57 *	0.30
	65. Enhance learning how to read	3.81	1.03	4.08	0.89	−2.33 *	0.28
	66. Encourage leisure and entertainment	3.49	1.05	4.14	1.01	−5.37 ***	0.63
	71. They are a motivating tool	4.25	0.84	4.52	0.72	−2.94 **	0.35
**D2**	73. Develop communication	3.78	0.97	4.03	0.83	−2.37 *	0.28
	74. Develop oral language	3.60	1.06	3.86	0.92	−2.19 *	0.26
	75. Develop the understanding of emotions	3.50	1.06	3.88	0.93	−3.19 **	0.38
	76. Develop the expression of emotions	3.45	1.04	3.86	0.97	−3.41 **	0.41

Note. *M* = Mean; *SD* = Standard Deviation; *t* = Student’s *t*, *d* = Cohen’s *d*, * *p* < 0.05, ** *p* < 0.01, *** *p* < 0.001.

**Table 7:** Significant differences in the benefits and uses of apps as a function of sex.

City	Dependent Variables	Men		Women		*t*	*d*
		*M*	*SD*	*M*	*SD*		
Florence	84. Hold attention for longer	3.62	0.96	4.15	0.85	−2.25 *	0.58
Granada	75. Develop the understanding of emotions	3.52	0.82	3.95	0.94	−2.13 *	0.49

Note. *M* = Mean; *SD* = Standard deviation; *t* = Student’s *t*, *d* = Cohen’s *d*, * *p* < 0.05.

**Table 8:** Significant differences in the benefits and uses of apps as a function of age.

Age Groups (Years Old)											
City	Dependent Variables	20–30		31–40		41–50		51–64		*F*	*η* ^2^
		*M*	*SD*	*M*	*SD*	*M*	*SD*	*M*	*SD*		
Florence	78. Autonomy	3.90	0.76	3.94	0.98	3.90	1.03	2.86	0.69	2.84 *	0.058
Granada	68. Effective intervention	4.24	0.82	3.77	1.03	4.12	0.87	3.76	0.94	2.89 *	0.019
	70. Consolidate concepts	4.52	0.61	4.15	0.83	4.22	0.82	4.05	0.74	2.90 *	0.046
	73. Communication	4.16	0.79	3.74	0.90	4.27	0.74	3.90	0.77	3.37 *	0.007
	79. Planning	3.96	0.86	3.62	1.05	4.00	0.81	3.43	0.87	2.98 *	0.035
	80. Organization	3.96	0.97	3.66	0.98	3.98	0.72	3.38	0.92	2.88 *	0.045
	81. Self-regulation	4.02	1.02	3.47	1.04	3.93	0.85	3.48	0.93	3.60 *	0.049

Note. *M* = Mean; *SD* = Standard deviation; *F* = ANOVA, *η*^2^ = eta squared, * *p* < 0.05.

**Table 9:** Significant differences in the benefits and uses of apps as a function of years of experience.

Years of Experience (Florence)										
Dependent Variables	≤5 Years		6–10 Years		11–20 Years		21–30 Years		*F*	*η* ^2^
	*M*	*SD*	*M*	*SD*	*M*	*SD*	*M*	*SD*		
73. Develop communication	3.87	0.94	3.39	0.96	5.00	0.00	4.00	1.00	3.83 *	0.019

Note. *M* = Mean; *SD* = Standard deviation; *F* = ANOVA, *η*^2^ = eta squared, * *p* < 0.05.

**Table 10:** Significant differences in the benefits and uses of apps as a function of the educational stage taught by the teachers from Florence.

Educational Stages Worked in											
Dependent Variables		Preschool (*n* = 14)		Primary (*n* = 32)		Preschool and Primary (*n* = 36)		Secondary (*n* = 45)		*F*	*η* ^2^
		*M*	*SD*	*M*	*SD*	*M*	*SD*	*M*	*SD*		
**D1**	65. Reading	3.14	1.51	3.81	0.90	4.06	0.96	3.82	0.94	2.757 *	0.100
	70. Consolidate	4.07	0.92	4.19	0.74	4.42	0.65	3.93	0.69	3.109 *	0.026
	72. Socialize	3.64	1.08	3.38	0.83	3.86	0.93	3.29	0.92	2.937 *	0.012
**D2**	82. Self-regulation	3.00	1.36	3.69	0.93	3.94	1.07	3.82	0.94	3.029 *	0.066
	83. Calculus	3.00	1.36	3.72	0.85	4.06	0.92	3.87	0.89	4.301 **	0.067
	84. Attention	4.21	1.05	4.13	0.85	4.50	0.66	3.71	0.92	6.223 **	0.069

Note. *M* = Mean; *SD* = Standard deviation; *F* = ANOVA, *η*^2^ = eta squared, * *p* < 0.05, ** *p* < 0.01.

**Table 11:** Significant differences in the benefits and uses of apps as a function of the educational stage taught by teachers from Granada.

Educational Stages Taught													
Dependent Variables		Preschool (*n* = 18)		Primary (*n* = 56)		Preschool and Primary (*n* = 71)		Secondary (*n* = 9)		Adults (*n* = 5)		*F*	*η* ^2^
		*M*	*SD*	*M*	*SD*	*M*	*SD*	*M*	*SD*	*M*	*SD*		
**D1**	64. Memory	4.28	0.83	4.18	0.86	4.15	0.92	3.33	1.00	4.80	0.45	2.715 *	0.006
	66. Leisure	4.06	0.94	4.45	0.66	4.01	1.17	3.33	1.19	4.40	0.89	3.328 *	0.022
	71. Motivation	4.56	0.62	4.57	0.66	4.54	0.74	3.78	0.97	5.00	0.00	3.216 *	0.008
**D2**	83. Calculus	3.78	1.01	3.78	0.88	4.00	0.96	2.89	1.36	4.60	0.55	2.843 *	0.027
	84. Attention	4.28	0.85	4.45	0.63	4.20	0.80	3.67	1.12	4.80	0.45	2.981 *	0.027

Note. *M* = Mean; *SD* = Standard deviation; *F* = ANOVA, *η*^2^ = eta squared, * *p* < 0.05.

**Table 12:** Significant differences in the benefits and uses of apps as a function of the work location of teachers from Florence and Granada.

**Work Location, Florence**									
**Dependent Variables**		**Urban** **(*n* = 19)**		**Rural** **(*n* = 95)**		**Both** **(*n* = 13)**		***F***	***η* ^2^**
		***M***	***SD***	***M***	***SD***	***M***	***SD***		
**D1**	72. Enhance socialization	3.89	0.88	3.39	0.95	3.85	0.80	3.309 *	0.007
**D2**	73. Develop communication	4.21	0.71	3.65	1.03	4.08	0.49	3.449 *	0.025
	76. Develop the expression of emotions	3.89	0.81	3.31	1.09	3.85	0.69	3.725 *	0.020
**Work Location, Granada**									
**Dependent Variables**		**Urban** **(*n* = 110)**		**Rural** **(*n* = 44)**		**Both** **(*n* = 5)**		***F***	***η* ^2^**
		***M***	***SD***	***M***	***SD***	***M***	***SD***		
**D1**	69. A complement for reinforcing	4.29	0.78	3.95	1.10	5.00	0.00	4.404 *	0.040

Note. *M* = Mean; *SD* = Standard deviation; *F* = ANOVA, *η*^2^ = eta squared, * *p* < 0.05.

**Table 13:** Significant differences in the benefits and uses of apps as a function of the teaching position of the educators from Florence.

Type of Teacher, Florence									
Dependent Variables		General (*n* = 33)		Support (*n* = 71)		Both (*n* = 22)		*F*	*η* ^2^
		*M*	*SD*	*M*	*SD*	*M*	*SD*		
**D2**	74. Develop oral language	3.97	0.85	3.61	1.01	3.14	1.25	4.470 *	0.71

Note. *M* = Mean; *SD* = Standard deviation; *F* = ANOVA, *η*^2^ = eta squared, * *p* < 0.05.

**Table 14:** Significant differences in the benefits and uses of apps as a function of the teaching position of the educators from Granada.

Type of Teacher, Granada							
Dependent Variables		General (*n* = 30)		Support (Therapeutic pedagogy) (*n* = 55)		*t*	*d*
		*M*	*SD*	*M*	*SD*		
**D2**	82. Enhance learning how to read	4.20	0.81	3.73	1.06	2.13 *	0.50

Note. *M* = Mean; *SD* = Standard deviation; *t* = Student’s *t*, *d* = Cohen’s *d*, * *p* < 0.05.

**Table 15:** Quality, specialized apps for people with autism used by the teachers from Florence and Granada.

App	Languages of the App				Florence *N* (%)	Granada *N* (%)
	Spanish	Italian	English	Other		
1. #Soyvisual	X				1 (0.8)	80 (50.3)
2. Otsimo Juegos de educación especial para niños	X		X		3 (2.4)	11 (6.9)
3. MITA: Language and Cognitive Therapy	X	X	X		27 (21.3)	32 (20.1)
4. Smile and Learn: Juegos educativos para niños	X	X	X		5 (3.9)	73 (45.9)
5. CPA: Comunicador personal adaptable	X				2 (1.6)	6 (3.8)
6. Symbotalk—AAC Talker	X	X	X		18 (14.2)	45 (28.3)
7. Michelzhino—emoçoes e autismo				X	1 (0.8)	4 (2.5)
8. Visual schedules and Social stories			X		4 (3.1)	10 (6.3)
9. LEA: lecto escritura para autismo	X				2 (1.6)	69 (43.4)
10. Autastico			X		2 (1.6)	10 (6.3)
11. Juego de niños para bebés de 2 a 5 años			X		4 (3.1)	38 (23.9)
12. Terapia Z Tabletem			X		1 (0.8)	6 (3.8)
13. ¡Emociones, sentimientos y expresiones!			X		4 (3.1)	57 (35.8)
14. Commboards: gratis terapia del autismo AAC	X		X		4 (3.1)	9 (5.7)
15. Proyecto emociones	X				3 (2.4)	44 (27.7)
16. Aboard CAA (ACC)				X	26 (20.5)	23 (14.5)
17. Social skills for autism Kloog2			X		2 (1.6)	6 (3.8)
18. José Aprende	X				0 (0.0)	69 (43.4)
19. Proyect@ PECS			X		8 (6.3)	40 (25.2)
20. Vi.co hospital Lite	X	X	X		13 (10.2)	4 (2.5)
21. Lista visual -Visual Schedule	X	X	X		0 (0.0)	21 (13.2)
22. ABA DrOmnibus for Parents			X		10 (7.9)	10 (6.3)
23. Speech Blubs: Language Therapy			X		13 (10.2)	18 (11.3)

**Table 16:** Statistically significant differences in the use of specialized apps according to the city of origin.

Name of the App	Florence		Granada		*t*	*d*
	*M*	*SD*	*M*	*SD*		
1. #Soyvisual	0.01	0.09	0.50	0.50	−10.99 ***	1.36
4. Smile and Learn: Juegos educativos para niños	0.40	0.20	0.46	0.50	−8.93 ***	0.16
6. Symbotalk—AAC Talker	0.14	0.35	0.28	0.45	−2.90 **	0.35
9. LEA: lecto escritura para autismo	0.02	0.13	0.43	0.50	−9.25 ***	1.12
10. Autastico	0.02	0.13	0.06	0.24	−1.98 *	0.21
11. Juego de niños para bebés de 2 a 5 años	0.03	0.18	0.24	0.43	−5.13 ***	0.64
13. ¡Emociones, sentimientos y expresiones!	0.03	0.18	0.36	0.48	−7.28 ***	0.91
15. Proyecto emociones	0.02	0.15	0.28	0.45	−6.08 ***	0.78
18. José Aprende	0.00	0.00	0.43	0.50	−9.83 ***	1.22
19. Proyect@ PECS	0.06	0.24	0.25	0.44	−4.37 ***	0.54
20. Vi.co hospital Lite	0.10	0.30	0.03	0.16	2.77 **	0.29
21. Lista visual -Visual Schedule	0.00	0.00	0.13	0.34	−4.38 ***	0.54

Note. *M* = Mean; *SD* = Standard deviation; *t* = Student’s *t*, *d* = Cohen’s *d*, * *p* < 0.05, ** *p* < 0.01, *** *p* < 0.001.
